# A Case of Type I Sirenomelia Complicated by Severe Oligohydramnios in the First Trimester

**DOI:** 10.1155/2019/4564260

**Published:** 2019-12-26

**Authors:** Atsushi Yoshida, Asumi Okumura, Masahiro Nakao, Ryo Suzuki

**Affiliations:** Department of Obstetrics and Gynecology, Sakakibara Heart Institute, Fuchu, Tokyo, Japan

## Abstract

Sirenomelia is a very rare congenital anomaly. Type I is the mildest type, and the long bone structures are all normally present with only soft tissue fusion. We experienced a case of type I sirenomelia complicated by severe oligohydramnios. Because of severe oligohydramnios, ultrasonographic images were not very clear. The associated findings with sirenomelia (single umbilical artery and bilateral renal agenesis) were helpful for the prenatal diagnosis of this disease. Detailed sonographic examination of the fetus was thought to be necessary for the accurate prenatal diagnosis of sirenomelia.

## 1. Introduction

Sirenomelia is a very rare congenital anomaly, and its incidence is reported to be 0.8 to 1 case per 100,000 births [1]. Sirenomelia is characterized by various kinds of fusions of the lower extremities and usually complicated by dysgenesis or agenesis of the kidneys, ureters, and urinary bladder. In most cases, it is also associated with single umbilical arteries. Because of the dysgenesis of the urinary system, sirenomelia is usually complicated by severe oligohydramnios in the second and third trimesters of gestation. Oligohydramnios may cause poor visualization in the ultrasonographic images, and it is not easy to make a diagnosis of sirenomelia in the second and third trimesters. Therefore, it is very difficult to diagnose sirenomelia with oligohydramnios, especially in cases with the normal number of long bones. We experienced a case of type I sirenomelia complicated by severe oligohydramnios at 13 weeks of gestation. We consider that this is the first report on type I sirenomelia with severe oligohydramnios diagnosed in the first trimester.

## 2. Case Presentation

**Figure 1:**
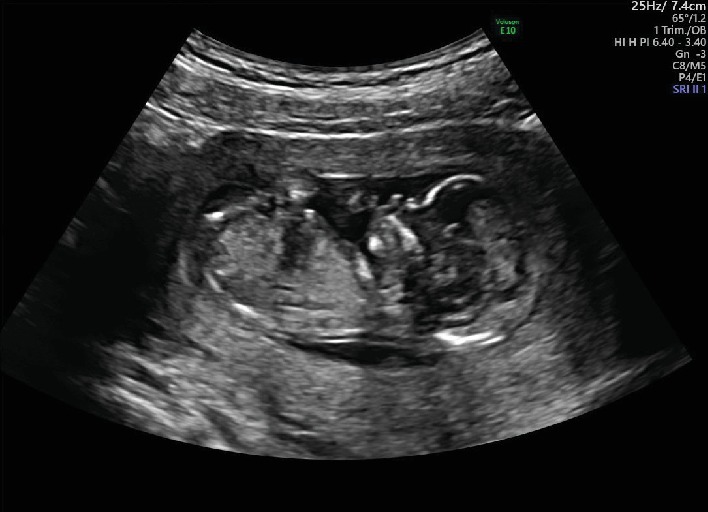
Gray-scale ultrasonography at the 13 weeks of gestation. Severe oligohydramnios was noted.

**Figure 2:**
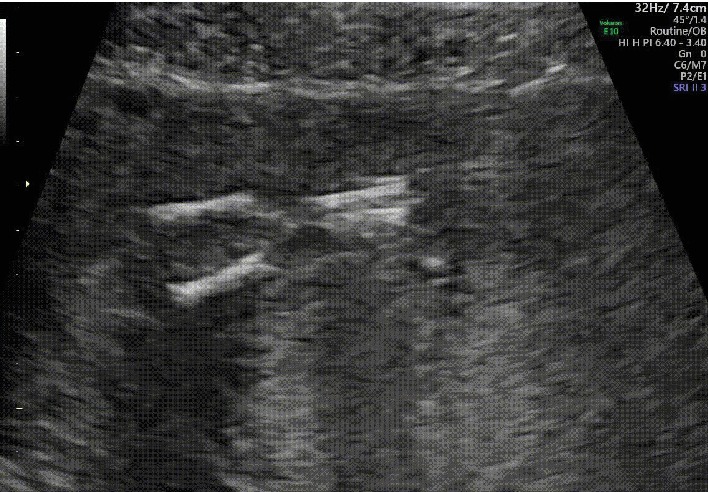
Gray-scale ultrasonography at the 14 weeks of gestation. Two femurs, two tibiae, and two fibulae were visualized.

**Figure 3:**
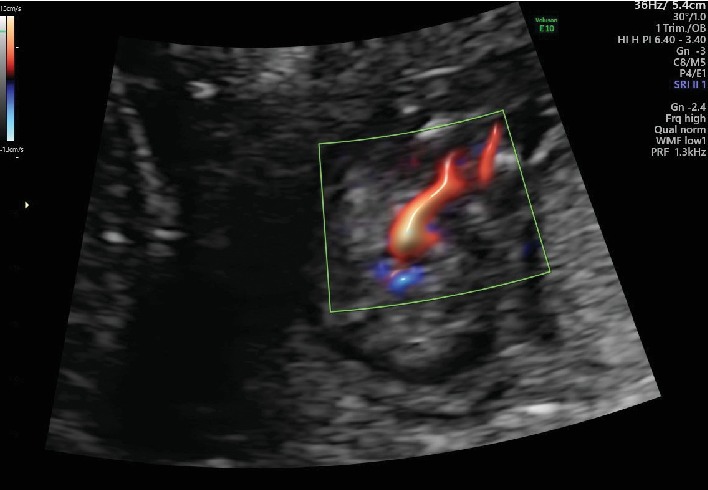
Color Doppler ultrasonography at the 13 weeks of gestation. A single umbilical artery was seen.

The patient was a 22-year-old nulliparous Japanese woman. Her medical and family histories were noncontributory. She had no previous history of smoking, habitual alcohol drinking, or drug abuse. She was referred to our hospital for fetal anatomical evaluation because of severe oligohydramnios at 13 weeks and 6 days of gestation. The gestational age was calculated from the first day of her last menstrual period. At her 10 weeks and 6 days' visit to the previous clinic, the crown-rump length of the fetus was 34.7 mm and no remarkable abnormalities of amniotic fluid volume were recorded. However, at 13 weeks and 4 days of gestation, a remarkable decrease in the amniotic fluid volume was found. At the first visit to our hospital, we carried out an antenatal ultrasonographic scan and a single live fetus was noted. The crown-rump length, biparietal diameter, and nuchal translucency of the fetus were 66.1 mm, 23.6 mm, and 2.1 mm, respectively. Because of severe oligohydramnios (Figure 1), ultrasonographic images were not very clear, and no clear 3D images were obtained. As far as we could check, no structural abnormalities were found in the fetal cranium, brain, upper extremities, thorax, and heart. However, the lower extremities remained in the fixed extension, but all long bones were visible (Figure 2). The lower extremities stayed in the same position, and no individual movements of the two legs were seen, which suggested the fusion of the two lower extremities. Color Doppler ultrasound revealed a single umbilical artery (Figure 3). Neither kidneys nor the urinary bladder was visualized. Therefore, the diagnosis of type I sirenomelia was made prenatally.

**Figure 4:**
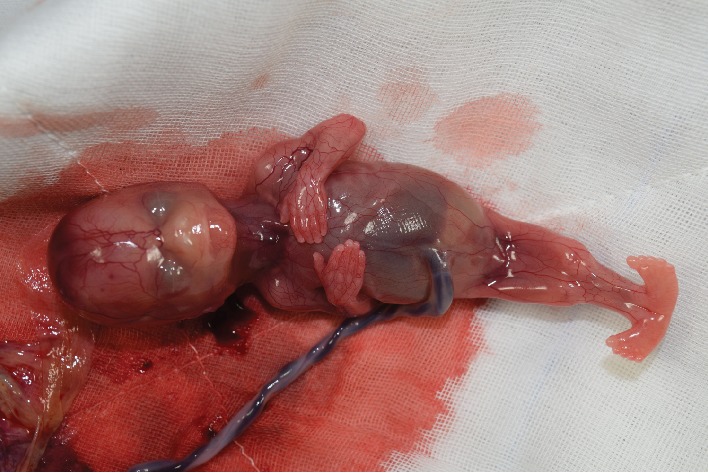
Macroscopic image of the delivered baby. A fused single lower extremity was noted.

**Figure 5:**
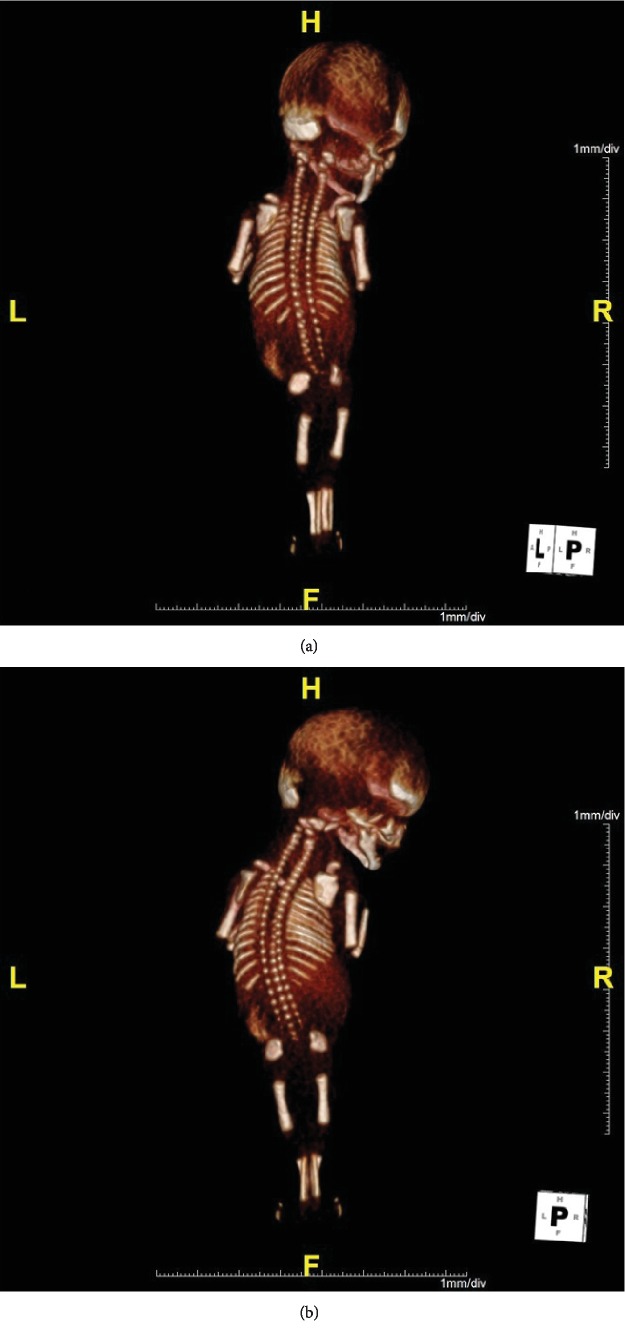
Postmortem CT images of the delivered baby. Two femurs, two tibiae, and two fibulae were contained in the single lower limb, and none of them was fused (a). Agenesis of lower lumbar and sacral spines was noted (b).

The couple was informed about the findings and the suspected poor prognosis of the fetus. They then opted to have a termination of pregnancy, which was performed at 15 weeks and 1 day of gestation. On the postnatal examination, the baby weighed 60 grams and the external examination revealed the fusion of both lower extremities (Figure 4). The external genitalia and anal structures of the baby were ambiguous. Postmortem imaging studies with the radiography and CT scans revealed a single lower limb containing two femurs, two tibiae, and two fibulae, and none of them were fused (Figure 5(a)). Agenesis of the lower lumbar and sacral spines was also noted (Figure 5(b)).

**Figure 6:**
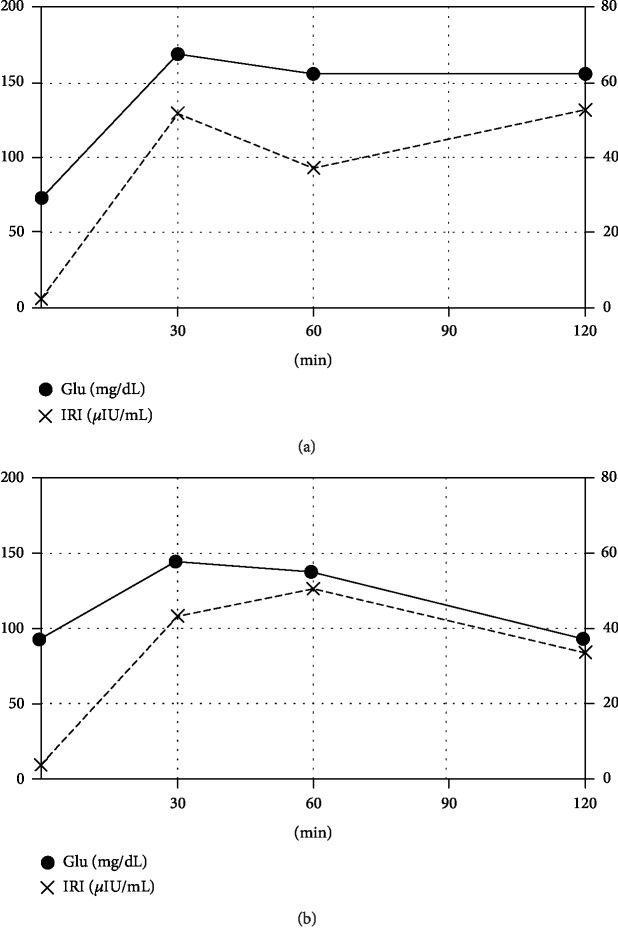
Results of the 75 g oral glucose tolerance test. (a) Two days postpartum: HOMA − R = 0.47, QUICKI = 0.44, MI = 9.38, DI = 4.81, and II = 0.51. (b) Seventeen weeks postpartum: HOMA − R = 0.82, QUICKI = 0.40, MI = 8.02, DI = 6.08, and II = 0.76. HOMA-R: homeostasis model assessment insulin resistance; QUICKI: quantitative insulin-sensitivity check index; MI: Matsuda index; DI: disposition index; II: insulinogenic index.

A 75 g oral glucose tolerance test (OGTT) was performed 2 days postpartum, which showed a mildly impaired glucose tolerance pattern of the mother (Figure 6(a)). At 17 weeks postpartum, another OGTT was carried out and the blood glucose levels were in the normal range (Figure 6(b)).

## 3. Discussion

**Figure 7:**
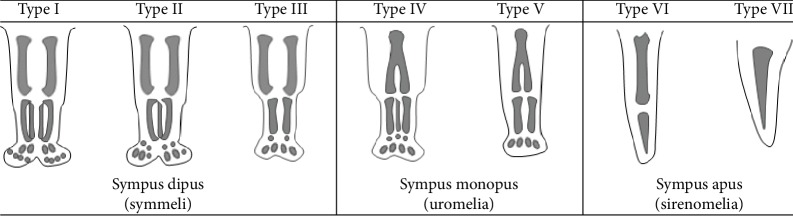
Classification of sirenomelia by Stocker and Heifetz [2]. Type I: all bones of thigh and lower leg present; type II: fused fibula; type III: fibula absent; type IV: partially fused femur, fused fibula; type V: partially fused femur, fibula absent; type VI: fused femur, fused tibia; type VII: fused femur, tibia absent.

Stocker and Heifetz classified sirenomelia into 7 types by the degree of fusion of lower extremities' bones (Figure 7) [2]. Type I is the mildest type, and the long bone structures are all normally present with only soft tissue fusion. We found no clear-cut data of the prenatal diagnostic rate by Stocker and Heifetz's classifications of sirenomelia, but Langer and colleagues reported that the difficulty of prenatal diagnosis depends on the shape of the lower extremity (the symelia apus, unipus, or dipus), and the dipus type is the most difficult to make a prenatal diagnosis [3]. Therefore, it is suspected that type I sirenomelia by Stocker and Heifetz's classifications is the least easy to make a prenatal diagnosis. In other types, the abnormality in the number of long bones may be a great help for the diagnosis of sirenomelia. In type I sirenomelia, however, it may be difficult to make a diagnosis by prenatal ultrasonography only with the abnormality in fetal soft tissue.

To detect the soft tissue abnormalities, 3D images of ultrasound are thought to be useful, but it is difficult to obtain clear 3D images in cases with anhydramnios or severe oligohydramnios. Langer and colleagues reported that it is difficult to make a prenatal diagnosis of this disease when complicated by severe oligohydramnios [3]. As far as we could check, we found no previous reports of type I sirenomelia with severe oligohydramnios diagnosed in the first trimester. In our case, we could not obtain clear 3D images of the fetus because of severe oligohydramnios. Some authors proposed to carry out amnioinfusion to obtain clearer ultrasound images when the case is complicated by severe oligohydramnios [4, 5]. However, we think that this invasive procedure is not necessary when the baby's prognosis is thought to be poor, and actually, in this case, we did not perform amnioinfusion.

The associated findings with sirenomelia may be helpful for the prenatal diagnosis of this disease.  Table 1 shows the common complications of sirenomelia [6]. In our case, agenesis of the lower lumbar and sacral spines and the presence of the single umbilical artery were noted. Bilateral renal agenesis was also suspected. These abnormalities have been suspected by prenatal ultrasound, and they were great help to diagnose sirenomelia.

As we described above, sirenomelia is often associated with agenesis or dysgenesis of bilateral kidneys. Even in the first trimester, it is possible to identify both kidneys with careful ultrasound examination, but recognizing oligohydramnios as a consequence of renal agenesis or dysgenesis in the second trimester is much easier. It is reported that usually, the onset of renal oligohydramnios starts after 16 weeks of gestation when amniotic fluid production is primarily renal in origin [7]. Therefore, the suspicion of renal agenesis before 15 weeks is sometimes challenging. Several cases with renal oligohydramnios before 15 weeks have been reported. Klaassen and colleagues reviewed 21 cases of renal oligohydramnios and reported that one of the 21 cases was detected at 14 weeks of gestation [8]. Ceylan and colleagues reported a case of sirenomelia diagnosed at 12 weeks of gestation [9], and they said that their case was complicated by oligohydramnios. However, they reported that they could obtain 3D ultrasound images of the fetus, which means that oligohydramnios in their case was not very severe. In our case, the cause of severe oligohydramnios at 13 weeks of gestation was not clear. It is said that the primary site of amniotic fluid production before 14 weeks of gestation is the amnion, but in our case, no pathological findings of the amnion were reported in the histopathological examination.

The causes of malformation in sirenomelia remain unknown, but it is reported that maternal diabetes mellitus is one of the possible causative factors of sirenomelia [10]. In our case, OGTTs were carried out 2 days and 17 weeks postpartum. The OGTT done after 2 days postpartum revealed a mildly impaired glucose tolerance pattern of the mother. However, the OGTT result after 17 weeks was normal. Therefore, we think that sirenomelia in our case was not directly caused by glucose intolerance. The mother had no other risk factors, and in this case, the etiology of sirenomelia remained unclear.

## 4. Conclusion

We experienced a case of type I sirenomelia complicated by severe oligohydramnios. Prenatal diagnosis of sirenomelia with severe oligohydramnios may be difficult, especially in type I sirenomelia in which all long bones are visible. The associated findings with sirenomelia may be helpful for the prenatal diagnosis of this disease. Detailed sonographic examination of the fetus was thought to be necessary for the prenatal diagnosis of sirenomelia.

## Figures and Tables

**Table 1:** Possible associated abnormalities with sirenomelia.

Urogenital
Renal agenesis
Multicystic dysplastic kidneys (rare)
Genital ambiguity/absence of external genitalia
Müllerian anomalies
Cloacal abnormalities
Gastrointestinal tract
Anorectal atresia
Sacral agenesis
Skeletal
Neural tube defects
Lumbosacral dysgenesis/agenesis
Sacral agenesis
Varying degrees of limb reduction, soft tissue fusion of lower extremities, single lower extremity
Hypoplasia/aplasia of pelvic girdle
Complex fusion of feet (sympodia)
Absent feet
Radial ray abnormalities
Phocomelia
Rotational abnormalities of lower limbs
Hip dislocation
Others
Single umbilical artery (virtually always present) Vestigial tail
Cardiac system anomalies (less common)
Central nervous system anomalies (less common)

^∗^Listed based on Reference [6].
